# Distinct subpopulations of human subcutaneous adipose tissue precursor cells revealed by single-cell RNA sequencing

**DOI:** 10.1152/ajpcell.00726.2023

**Published:** 2024-03-04

**Authors:** Adeline Divoux, Katie L. Whytock, Laszlo Halasz, Meghan E. Hopf, Lauren M. Sparks, Timothy F. Osborne, Steven R. Smith

**Affiliations:** ^1^Translational Research Institute, AdventHealth, Orlando, Florida, United States; ^2^Division of Diabetes Endocrinology and Metabolism, Johns Hopkins University School of Medicine, Institute for Fundamental Biomedical Research, Johns Hopkins All Children’s Hospital, St. Petersburg, Florida, United States; ^3^Department of Medicine, Johns Hopkins University School of Medicine, Institute for Fundamental Biomedical Research, Johns Hopkins All Children’s Hospital, St. Petersburg, Florida, United States; ^4^Department of Biological Chemistry, Johns Hopkins University School of Medicine, Institute for Fundamental Biomedical Research, Johns Hopkins All Children’s Hospital, St. Petersburg, Florida, United States; ^5^Department of Pediatrics, Johns Hopkins University School of Medicine, Institute for Fundamental Biomedical Research, Johns Hopkins All Children’s Hospital, St. Petersburg, Florida, United States

**Keywords:** abdominal adipose tissue, adipose-derived stem cells, cell identity, gluteofemoral adipose tissue, scRNA-seq

## Abstract

Adipose-derived stem cells (ADSCs) play an important role in the differential capacity for excess energy storage between upper body abdominal (ABD) adipose tissue (AT) and lower body gluteofemoral (GF) AT. We cultured ADSCs from subcutaneous ABD AT and GF AT isolated from eight women with differential body fat distribution and performed single-cell RNA sequencing. Six populations of ADSCs were identified and segregated according to their anatomical origin. The three ADSC subpopulations in GF AT were characterized by strong cholesterol/fatty acid (FA) storage and proliferation signatures. The two ABD subpopulations, differentiated by higher expression of committed preadipocyte marker genes, were set apart by differential expression of extracellular matrix and ribosomal genes. The last population, identified in both depots, was similar to smooth muscle cells and when individually isolated and cultured in vitro they differentiated less than the other subpopulations. This work provides important insight into the use of ADSC as an in vitro model of adipogenesis and suggests that specific subpopulations of GF-ADSCs contribute to the more robust capacity for GF-AT to expand and grow compared with ABD-AT in women.

**NEW & NOTEWORTHY** Identification of distinct subpopulations of adipose-derived stem cells (ADSCs) in upper body abdominal subcutaneous (ABD) and lower body gluteofemoral subcutaneous (GF) adipose tissue depots. In ABD-ADSCs, subpopulations are more committed to adipocyte lineage. GF-ADSC subpopulations are enriched for genes involved in lipids and cholesterol metabolism. Similar depot differences were found in stem cell population identified in freshly isolated stoma vascular fraction. The repertoire of ADSCs subpopulations was different in apple-shaped versus pear-shaped women.

## Graphical abstract

**Figure:**
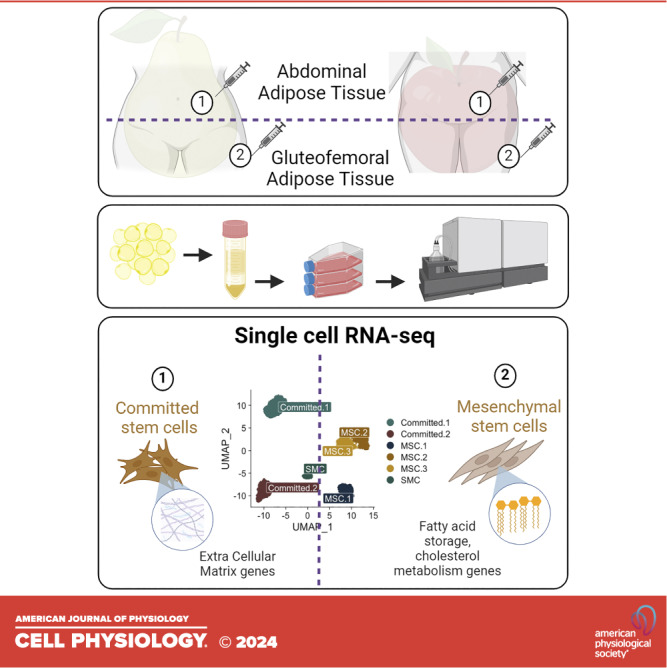

## INTRODUCTION

Subcutaneous adipose tissue (SAT) is involved in the development of the metabolic syndrome (MS) seen with central obesity (1, 2). Accumulation of both SAT and visceral adipose tissue (VAT) in the upper body (or above the waist) is associated with an increased risk of MS comorbidities, whereas excess SAT in the lower body is protective against the same risks (3–5). This inverse relationship suggests that adipose depots below the waist serve as a metabolically healthy lipid storage sink during periods of weight gain or alternatively secrete unidentified beneficial factor(s). Defining the fundamental differences between upper and lower body adipose tissues (ATs) is primordial to understanding their role in the whole body phenotype and their individual role in the development of MS. In addition, why some women accumulate fat preferentially in their upper body (apple-shaped) and others preferentially expand their lower body adipose depots [gluteal, gluteofemoral (GF), and thigh; pear-shaped] is still unknown.

Ninety percent of adipose tissue lipid mass is in the adipocytes but they only account for ∼40% of the total cells in the subcutaneous adipose depots (6). The majority of adipose depot cells are contained in the stromal vascular fraction (SVF), including but not limited to endothelial cells, immune cells, and adipocyte precursors (stem cells and preadipocytes). The latter have been intensively used in vitro to study AT biology, notably to understand the mechanisms of adipogenesis. In brief, the SVF is separated from mature adipocytes by collagenase digestion and low-speed centrifugation. When the SVF is cultured ex vivo, blood cells, endothelial cells, and other nonfibroblastic cells do not attach to the culture plastic flask. What remains (adipose-derived stem cells—ADSCs) can be almost completely differentiated to mature adipocytes using a hormonal cocktail that typically includes insulin, a glucocorticoid, a phosphodiesterase inhibitor, and a peroxisome proliferator-activated receptor (PPAR)γ agonist. Interrogating all ADSCs does not allow for the identification of specific cell type(s) within the SVF that contribute to the mature adipocyte fraction in vivo and therefore this has not helped to determine if the ADSCs isolated from abdominal (ABD) AT and GF AT have an intrinsic differential capacity for differentiation. To work around this issue, some studies have proposed to enrich selective subpopulations of ADSCs through fluorescence-activated cell sorting (FACS) (6–8). However, the significant downside of this method is the limited quantity of remaining cells for downstream analysis after antibody-directed cell sorting (often needs an extensive number of days in culture after sorting the cells).

To develop a different ex vivo model using a large number of adipocyte precursor cells, we leveraged single-cell RNA-seq to identify subpopulations of cultured ADSCs isolated from ABD and GF adipose tissues that we predict may contribute to differential adipose tissue distribution and associated metabolic complications. Single-cell (sc) and single-nuclei (sn) RNA-seq technologies have previously identified subpopulations of precursor cells in ABD-SAT and VAT in mice (9) and in humans (10, 11), potentially leading to functionally distinct populations of adipocytes (12–14). Importantly, cultured ADSCs isolated from the ABD- and GF SVF retain the depot-specific gene expression patterns (15–18), suggesting that the phenotypic and transcriptional differences between ABD and GF adipose tissue depots are at least partly cell autonomous. Here, we hypothesized that the repertoire and proportion of stem and committed cells in upper versus lower adipose tissue could independently influence adipose tissue expansion and function and that their unique transcriptome is maintained in culture.

## MATERIALS AND METHODS

### Participants and Tissue Collection

The method of recruitment, clinical, and biochemical parameters of subjects are presented in the study by Divoux et al. (19). Briefly, paired abdominal and gluteofemoral subcutaneous white adipose tissue samples were obtained from eight healthy premenopausal, weight-stable females. Four women display lower body adiposity characterized by a percentage of fat mass in the legs compared with the total fat mass ≥ 40% (pear group) and four women display upper body adiposity, characterized by a percentage of fat mass in the legs compared with the total fat mass < 40% (apple group). The clinical characteristics of the two groups are summarized in Supplemental Table S1. All procedures were performed under a research protocol approved by the AdventHealth Institutional Review Board and all participants provided informed written consent.

The adipocyte size (micrometers) was estimated on adipose tissue sections by measuring the major diameter of 200 cells from digital microscopic images using NIS-Elements AR5.02 (Nikon, Minato, Japan) software.

### Isolation of Human Adipose-Derived Stem Cells and In Vitro Experiments

Abdominal and gluteofemoral subcutaneous adipose tissues were minced and incubated with 1 mg/mL collagenase type I supplemented with 1% BSA in a shaking water bath at 37° for 45 min. To eliminate mature adipocytes, the obtained suspension was centrifuged (200 *g*, room temperature, 5 min). The remaining tissue fragments were eliminated through filtration of resuspended pellets on 100- and 40-μm strainers. Blood contamination was removed by incubating the pellet suspension in red blood cell lysis buffer for 5 min. The resulting cell suspension was depleted of endothelial cells using magnetic beads coupled to CD31 antibody (15) and the cells in the flow-through fraction were plated in tissue culture flasks with α Dulbecco’s modified Eagle’s Medium (αMEM) supplemented with 10% fetal bovine serum (FBS), 100 U/mL penicillin, and 100 μg/mL streptomycin, for adipose progenitor cells isolation by plastic adherence. After 24 h, the nonadherent cells were washed away by replacing the media with αMEM supplemented with 2.5% FBS, EGF (10 ng/mL), and FGF (1 ng/mL). Adherent cells were maintained at 37°C in a humid atmosphere with 5% CO_2_ until the monolayer of cells reached confluence, with medium change every 2 days. After confluence was reached at *passage 2*, adherent cells were harvested with 10% trypsin/EDTA, slow-frozen, and stored at −80°C. These cells, named adipose-derived stem cells (ADSCs) were assessed for mycoplasma contamination using a luciferase enzyme-based assay, the MycoAlert Detection kit (Lonza), according to the manufacturer’s instructions.

### Single-Cell RNA-Sequencing on ADSCs

One aliquot of ADSCs for each participant was thawed and cultured for 3 days or until 80% confluency. Cells were harvested and counted with a Countess II automated cell counter (Thermo Fisher Scientific). Single-cell suspension (56,000/mL) was distributed equally into eight wells of a 384-well source plate (Takara Bio USA, San Jose, CA) and dispensed onto an ICELL8 350v Chip (Takara Bio USA) using an ICELL8 MultiSample NanoDispenser (Takara Bio USA). Using the ICELL8 system allows for multiple samples to be loaded on the same chip while being able to bioinformatically retrieve which nuclei originate from which sample from known index primer combinations. To limit batch effects, we distributed four different subject samples on the same chip. The method used was as previously described (6). In brief, mRNA from single cells were isolated and converted to full-length cDNA before unbiased amplification of 3′ and 5′ ends using SMART-Seq ICELL8 Application Kit (Takara Bio USA). Multiplexed sequencing libraries were generated from cDNA using the Illumina Nextera XT protocol and 150-bp paired-end sequencing was performed on an Novogene HiSeqX instrument.

### Single-Cell RNA-Sequencing on Stroma Vascular Fraction

SVF was isolated from ABD- and GF adipose tissue biopsies as previously described (20) from five healthy women [38 yr ± 4.4; 31.8 kg/m^2^ ± 3.12; waist to hip ratio (WHR) 0.86 ± 0.05]. After isolation, cells were counted with a Countess II automated cell counter (Thermo Fisher Scientific). Single-cell suspension (56,000/mL) was distributed into eight wells of a 384-well source plate (Takara Bio USA, San Jose, CA) and dispensed onto an iCELL8 350v Chip (Takara Bio USA) using an iCELL8 MultiSample NanoDispenser (Takara Bio USA). We distributed the ABD- and GF SVF of each subject on the same chip. The method used was as previously described (6). In brief, mRNA from single cells were isolated and converted to full-length cDNA before unbiased amplification of 3′ and 5′ ends using SMART-Seq ICELL8 Application Kit (Takara Bio USA). Multiplexed sequencing libraries were generated from cDNA using the Illumina Nextera XT protocol and 150-bp paired-end sequencing was performed on a Novogene HiSeqX instrument.

### Bioinformatics

Demultiplexing, mapping, alignment, and counting of the single-cell libraries were performed using CogentAP Analysis Pipeline (Takara Bio USA), using GRCh19 as a genome reference. Cell and gene filtering and clustering were performed in R package Seurat version 4.3.1 (21). Cells were removed if they had *1*) <1,500 genes, *2*) >20,000 genes (for ADSC) or >30,000 genes (for SVF cells), *3*) >20% of mitochondrial reads for ADSCs or >30% of mitochondrial reads for SVF cells. Data were log normalized and 2,000 highly variable genes were selected with “versus” method for clustering. Sample integration and batch correction between the four separate chips were performed with different R packages: fastMNN, STACAS, RPCA, and Harmony (22) (Supplemental Fig. S1*A*). Harmony was chosen as the optimal integration tool because using this method, each participant was represented in each cell cluster as anticipated, and transcriptional differences between depots were apparent (23). Integrated clusters were visualized with UMAP. Individual UMAP (per subject) are shown in Supplemental Fig. S1*B*. Differential gene expression analysis was performed using a Wilcoxon rank sum test with Seurat’s “FindMarkers” function. A hypergeometric test was used to assess over-representation of upregulated genes (log_2_ FC > 0.25) from each cluster using the hypeR package and the Hallmark database (24, 25).

### Validation Studies

For validation studies, we used ADSCs isolated from ABD- and GF ATs as described earlier. Cells were FACS sorted using a BD FACS ARIA SORP cell sorter (FACSDiva software v.5, BD Biosciences, San Jose, CA) equipped with two lasers (blue 488 nm and red 640 nm) as previously described (26). All antibodies were mouse monoclonal and specific against human cell surface markers. We performed four independent experiments to isolate the following subpopulations: mesenchymal stem cell (MSC)-1, anti-CD90 PE Cyanine7 (clone 5E10, BD) and anti-CD151 APC (clone CB-CALLA); MSC-2, anti-CD44 APC (clone IM7, BD); smooth muscle cell (SMC), anti-CD59 APC (clone OV9A2, BD). Expression of these four genes in the six clusters of ADSCs is shown in Supplemental Fig. S4. After sorting, the cells were plated, and cultured in αMEM/2.5% FBS media supplemented with hEGF and hFGF (10 μg/mL and 4 μg/mL, respectively). At confluence, the cells were frozen in αMEM/10% DMSO and preserved in LN_2_. One vial of each subpopulation was thawed to perform the following assays: *1*) cells were plated, grown, and differentiated in DMEM/F12 medium (final concentration of 50 nM insulin, 100 nM dexamethasone, 0.25 mM inhibitor 1-methyl-3-isobutylxanthine, and 100 nM rosiglitazone) for 4 days. Next, this medium was replaced by DMEM/F12 (final concentration of 50 nM insulin and 100 nM rosiglitazone). The medium was changed every 2 days until 12 days. Cells were harvested before and after differentiation (*D0* and *D12*), RNA was extracted, cDNA was generated by reverse transcriptase, and target genes were measured by real-time RT-PCR using a ViiA7 sequence detection system (Life Technologies) and SYBRGreen technology suitable for relative gene expression quantification using the following parameters: one cycle of 95°C for 10 min, followed by 40 cycles at 95°C for 15 s and 60°C for 1 min. Cyclophilin A (*PPIA*) was used as a control housekeeping gene and delta-delta Ct method was used to calculate relative gene expression values. *2*) Cells were plated, grown, and differentiated as described earlier. Cells were harvested before and after differentiation (*D0* and *D12*) in RIPA buffer with a protease inhibitor cocktail. Equal amounts of total protein were resolved in 4%–20% Tris-HCl gels (BioRad) and transferred to PVDF membranes. Membranes were probed for SCD1 and Hsp90. Chemiluminescence images were captured using the Odyssey CLx imager (LI-COR Biosciences).

## RESULTS

**Figure 1.**
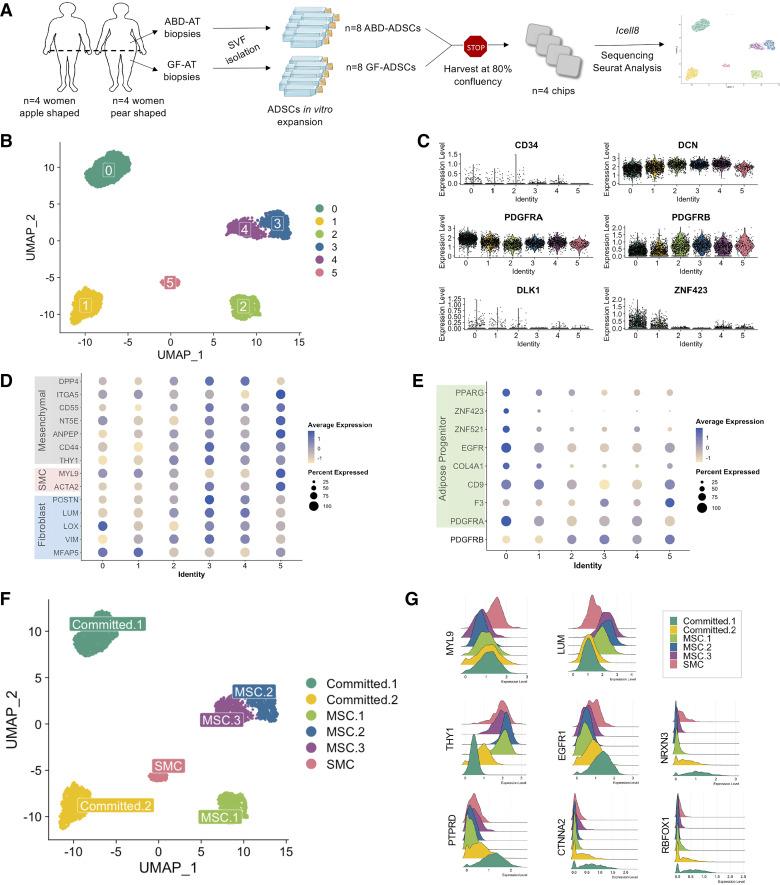
A single-cell atlas of human culture adipose-derived stem cells (ADSCs) isolated from abdominal (ABD) and gluteofemoral (GF) adipose tissue (AT). Schematic of workflows used in the study of ADSCs isolated from abdominal (ABD) and gluteofemoral (GF) adipose tissue (AT), created with the material from Servier Medical Art https://creativecommons.org/licenses/by/4.0/ (*A*). UMAP showing six clusters from 4,182 ADSCs (*B*). Violin plots showing stem cells and committed preadipocyte marker genes in the 6 clusters (*C*). Dot-plot showing fibroblast, smooth muscle cell (SMC) and mesenchymal stem cell (MSC) marker genes expression in the 6 clusters (*D*). Dot-plot showing committed preadipocyte marker genes in the six clusters (*E*). UMAP showing the six clusters named (*F*). Ridge plot showing expression of marker genes used to label the 6 clusters (*G*). SVF, stroma vascular fraction.

Using ICELL8 Smartseq full length technology, we performed single-cell RNA sequencing (scRNA-seq) on cultured adipose-derived stem cells (ADSCs) isolated from paired abdominal (ABD) and gluteofemoral (GF) subcutaneous adipose tissues from eight healthy premenopausal women (Fig. 1*A*). Four women had an apple-shaped fat distribution pattern defined by a percentage of fat mass in the leg relative to total fat mass [measured by dual X-ray absorptiometry (DEXA)] less than or equal to 40% (27). The other four women had a pear-shaped fat distribution pattern. To minimize variability from each library preparation, we loaded four samples on each chip (Fig. 1*A*). Clinical and adipose tissue characteristics of the participants are summarized in Supplemental Table S1. To remove the degree of obesity as a confounding factor, both groups were matched for age, body mass index (BMI), and percentage of total fat mass. All participants were clinically healthy with no overt signs of metabolic syndrome. However, as previously reported (19), the apple-shaped women were beginning to develop signs of metabolic disease with increased levels of circulating triglycerides, visceral fat mass, and liver fat compared with the pear-shaped subjects (Supplemental Table S1). These observations are consistent with our overall hypothesis: a defect in lipid storage in the subcutaneous adipose tissue depots of the apple-shaped women lies upstream of obesity-related metabolic complications (28).

### A Single-Cell Atlas of Human Subcutaneous WAT-ADSCs

In total, 4,317 sequenced ADSCs were included in our analysis. After appropriate quality filtering (see materials and methods), we generated a library of 4,182 single cells (2,256 ABD-ADSCs and 1,926 GF-ADSCs). Unsupervised clustering of the 4,182 ADSCs revealed six uniquely defined clusters (Fig. 1*B*). A majority of the expected mesenchymal stem cell (MSC) marker genes were detected in each cluster (e.g., *DCN*, *PDGFRA* and *PDGFRB*, Fig. 1*C*), except for CD24 (not expressed) and CD34, which was expressed at a very low level (Fig. 1*C*), suggesting that culture conditions may have resulted in loss of expression of some mesenchymal marker genes. Similar observations have been previously reported (29), confirming the need to use an unbiased approach to study cultured ADSCs instead of marker-based selection.

As expected, we detected the expression of fibroblast marker genes (*MFAP5*, *VIM*, *LOX*, *LUM*, *POSTN*) in varying degrees across the cell clusters (Fig. 1*D*). These genes have been previously shown to be expressed by adipose tissue progenitor cells (30–33). The detection of the known smooth muscle cell (SMC) marker genes *ACTA2*, *MYL9* in *cluster 5* suggests the presence of SMC within the cultured ADSCs (34) (Fig. 1*D*). Using markers identified previously by scRNA-seq (11, 35) or by FACS (36, 37), we classified three of the five remaining clusters as more primitive “MSC” (*clusters 2*, *3*, and *4*) (combination of *THY1*, *CD44*, *ANPEP*, *NT5E*, *CD55*, *ITGA5*; Fig. 1*D*) and the two other clusters were classified as more committed into the adipocyte lineage. These two clusters expressed the highest number of preadipocyte marker genes (including *CD36, COL4A1*, *EGFR*, *ZNF521, ZNF423,* and *PPARG—*Fig. 1*D*) (*clusters 0* and *1*). The violin plots in Fig. 1*C* confirm that *DLK1* and *ZNF423*, critical transcriptional regulators in adipose lineage commitment, were expressed at higher levels in some of the cell clusters including *clusters 0* and *1* suggesting that these clusters contained the most committed ADSCs of all the identified clusters (Fig. 1*C*).

Interestingly, the two committed subpopulations expressed higher levels of *PDGFRA*, whereas the more primitive populations expressed higher levels of *PDGFRB*, consistent with a recent publication showing that *PDGFRB* precedes *PDGFRA* expression during adipogenic commitment (38).

Based on the differentially enriched gene signatures, we renamed *clusters 0* and *1* “Committed-1” and “Committed-2”, the *clusters 2*, *3*, and *4* “MSC-1,” “MSC-2,” and “MSC-3,” and *cluster 5* “SMC” (Fig. 1*F*). The plot Fig. 1*G* depicts the expression in the six ADSC clusters of known SMC marker gene (*MYL9*), fibroblast (*LUM*), MSC (*THY1*), and preadipocytes (*EGFR1*) (39). We also found higher expression of four recently identified committed preadipocyte marker genes (*NRXN3*, *PTPRD*, *CTNNA2*, *RBFOX1*), in the committed ADSC subpopulations compared with MSC and SMC subpopulations (Fig. 1*G*). These markers were identified by snRNA-seq in whole human adipose tissue (40), showing that marker genes highlighted in vivo were maintained in culture.

### Identification of Depot-Specific ADSC Populations

**Figure 2.**
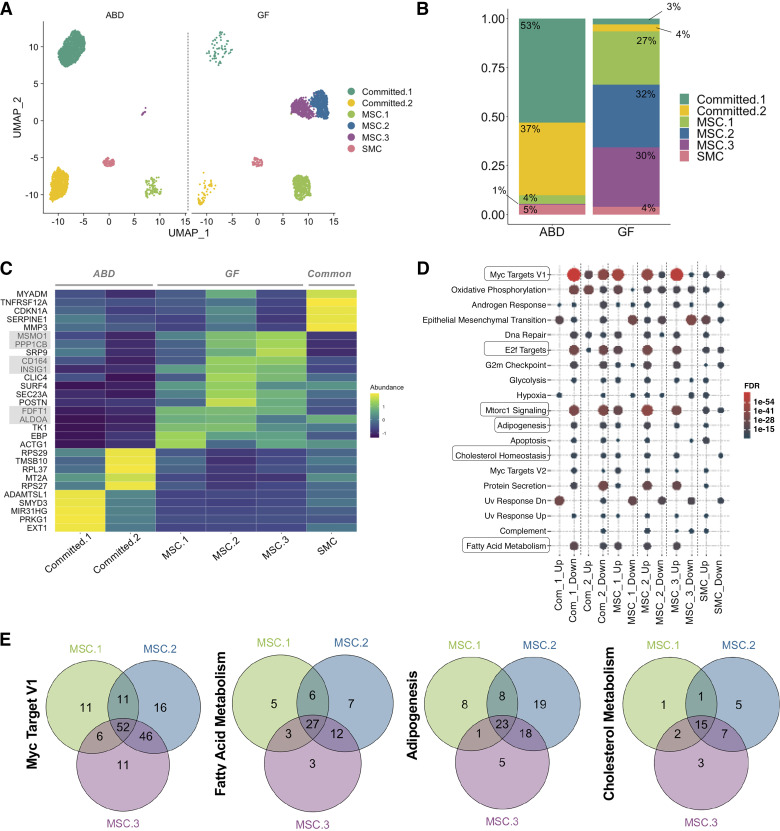
Depot-specific adipose-derived stem cell (ADSC) subpopulations. UMAPs and stacked bar-plots showing the repartition of the ADSCs subpopulations according to the adipose tissue depot [abdominal (ABD) vs. gluteofemoral (GF)] (*A* and *B*). Heatmap representation of the top five most upregulated genes (higher fold change) in each cluster of cells (*C*). Dotplot showing the significant pathways that are upregulated in each cluster; selected pathways with FDR < 0.05 are represented (*D*). Venn diagrams showing the common and unique genes differentially expressed in mesenchymal stem cell (MSC)-1, MSC-2, and MSC-3 and belonging to “Myc Target V1,” Fatty acid metabolism,” “Adipogenesis,” and cholesterol metabolism” pathways (*E*). SMC, smooth muscle cell.

We separated the ADSC data according to their anatomical origin and interestingly, we found a strong depot-specific association for the relative proportion of different ADSC clusters. The clusters labeled “Committed” were primarily from ABD ADSCs; whereas the “MSC” clusters were primarily from GF ADSCs (Fig. 2*A*). ABD- and GF ADSCs included the same proportion of SMC (5% and 4%, respectively; Fig. 2*B*).

A heatmap representation of the five top most upregulated genes in each cluster indicates that the ABD clusters were clearly transcriptionally distinct from each other. In contrast, the GF clusters appear more transcriptionally similar to each other (Fig. 2*C*). Importantly, genes related to lipid and cholesterol metabolism were upregulated in GF clusters (*MSMO1*, *INSIG1*, *FDFT1*, *ALDOA*, and *EBP*). Two genes coding for potential adipogenic activators [*PPP1CB* (41), and *CD164* (42)] were upregulated in all three MSC clusters and at higher levels in MSC-2 and MSC-3. *EXT1*, which encodes an enzyme essential for heparan sulfate biosynthesis, was the highest upregulated gene in the Committed-1 cluster. Importantly, heparan sulfate promotes differentiation of white adipocytes (43). *TMSB10*, whose expression positively correlated with sprouting of adipose tissue in an ex vivo model of adipose tissue expansion (44), and this gene was one of the most upregulated genes in the Committed-2 subpopulation. Finally, *CDKN1A*, a master regulator of cell cycle progression at G1 phase, was one of the most highly expressed genes in SMC.

To further characterize the depot-specific subpopulations of ADSCs, we performed differential gene expression analysis. The total number of differentially expressed genes (or DEGs) in each cluster is depicted in Supplemental Fig. 2*A*. We analyzed the pathway distribution for the cluster-specific DEGs. The most significant pathways that were enriched in each cluster are summarized in Fig. 2*D.* The GF-specific subpopulations (MSC-1, MSC-2, and MSC-3) showed several overlapping upregulated pathways including an enrichment of genes involved in lipid metabolism [cholesterol homeostasis, fatty acid (FA) metabolism], glycolysis, adipogenesis, mTORC1 signaling, and cell growth (Myc Targets V1, protein secretion, G2M checkpoint, and E2F targets). Interestingly, the same pathways were downregulated in the ABD-specific clusters (Committed-1 and Committed-2). “Apoptosis” was upregulated in the SMC cluster, suggesting that these cells were in a terminal stage.

Interestingly, when we intersected the list of genes of “Myc Target V1,” “FA metabolism,” “Adipogenesis,” and “Cholesterol metabolism” pathways, we found a high number of genes belonging simultaneously to all three GF-specific clusters (MSC-1, -2, and -3) but also some genes uniquely differentially expressed in only one of the clusters (Fig. 2*E*), particularly in MSC-2.

**Figure 3.**
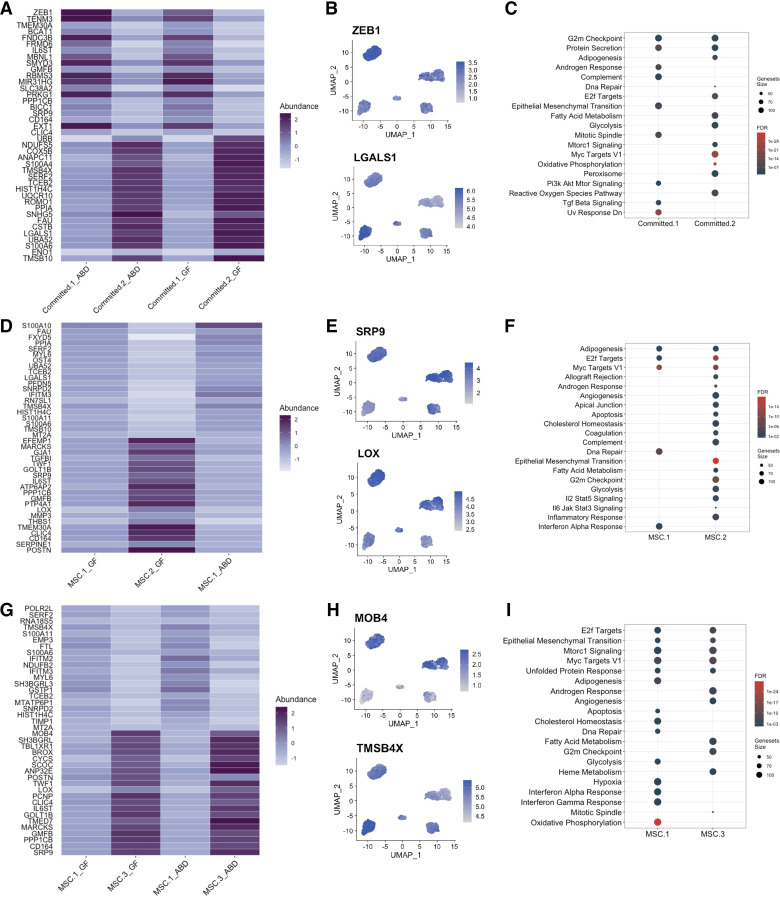
Unique transcriptome of depot specific adipose-derived stem cell (ADSC) subpopulations. Heatmap representation of the top 20 differentially expressed genes between Committed-1 and Committed-2 subpopulations in abdominal (ABD) and gluteofemoral (GF) depots (*A*). Feature plot showing expression of *ZEB1* and *LGALS1* genes (*B*). Dot-plot showing the significant pathways that are upregulated in each cluster, FDR < 0.05 (*C*). Heatmap representation of the top 20 differentially expressed genes between mesenchymal stem cell (MSC)-1 and MSC-2 subpopulations in ABD and GF depots (*D*). Feature plot showing expression of *POSTN* and *SRP9* genes (*E*). Dot-plot showing the significant pathways that are upregulated in each cluster, FDR < 0.05 (*F*). Heatmap representation of the top 20 differentially expressed genes between MSC-1 and MSC-3 subpopulations (*G*). Feature plot showing expression of *MOB4* and *TMSB4X* genes (*H*). Dot-plot showing the significant pathways that are upregulated in each cluster, FDR < 0.05 (*I*).

Next, we explored the differences between the two committed clusters, both ABD-specific by performing DEG analyses using only these two subpopulations. The heatmap in Fig. 3*A* depicts the most DEGs comparing Committed-1 and Committed-2 populations in both depots. This showed *ZEB1*, a central transcriptional component of fat cell differentiation (45), was upregulated in Committed-1 population, in both depots; whereas *LGALS1*, an activator of PPARG expression, was upregulated in Committed-2. We also found *CTSB* upregulated in Committed-2. *CTSB* codes for a lysosomal protease of which the expression and activity are impaired in adipose tissue of obese rodents and its expression is also associated with insulin resistance (46). Genes involved in cell proliferation (*TMSB4X* and *TMSB10*) were also upregulated in Committed-2. Pathway analysis revealed that Committed-2 was the most committed cluster to the adipocyte lineage reflected by upregulation of genes involved in pathways such as “Adipogenesis,” “FA metabolism,” “Mtorc 1,” and the most proliferative cluster (highlighted by increased “Myc Target V1” pathway). On the opposite, genes upregulated in Committed-1 are involved in “Epithelial-Mesenchymal Transition (EMT),” “TGFβ signaling,” and “Pi3k Akt mTOR signaling,” all of them potentially inhibitors of adipogenesis. We also noticed a high proportion of ribosomal (RB) genes being upregulated in Committed-2 cluster. We plotted all the ribosomal genes known to be involved in biological function [see Ref. (47), Supplemental Fig. S2*B*] and confirmed that Committed_2 was the cluster with the highest level of RB genes expression. Interestingly, only *RPL3*, known to prevent cell apoptosis, was downregulated. Posttranscriptional regulation by RB proteins is an essential part of adipogenesis (48). Altogether our observations suggest that Committed-2 cells are active in the process of differentiation from precursor to mature cells.

As a next step, we studied the differences between the three clusters enriched in GF, all of them being primitive stem cells (compared with the ABD clusters), by performing DEG analyses using only these three subpopulations and comparing them to each other one by one (Fig. 3, *D–I* and Supplemental Fig. S3). Genes involved in cell proliferation (*FAU*, *TMSB4X*, *TMSB10*, and *SRP9*) were upregulated in MSC-1 compared with MSC-2 (Fig. 3, *D* and *E*). On the opposite, *LOX*, known to promote adipogenesis through inhibition of the FGF-2 pathway (favoring proliferation) (49) was upregulated in MSC-2 (Fig. 3*E*). In addition, genes involved in extracellular matrix remodeling (*TGFB1*, *MMP3*, and *POSTN*) and inflammation (*IL6ST* and *SERPINE-1*) were upregulated in MSC-2 (Fig. 3, *D* and *E*). Pathway analysis between MSC-1 and MSC-2 showed that MSC-2 was enriched for genes involved in cholesterol homeostasis and FA metabolism along with genes involved in EMT and inflammatory responses (Fig. 3*F*). These two last pathways are induced when cells change their morphology such as it is observed during adipogenesis, transitioning from a fibroblastic-like morphology to the round morphology of an adipocyte. “Complement” pathway was also increased in MSC-2; complement components are involved in lipid metabolism. These results suggest that MSC-2 is more capable of both storage of lipids and adipocyte differentiation relative to MSC-1.

When comparing MSC-1 with MSC-3 subpopulations (Fig. 3, *G–I*), we found similar differences as between MSC-1 and MSC-2 (*TMSB4X* upregulated in MSC-1; Fig. 3*H*; and *POSTN*, *LOX*, *IL6ST* and *SRP9* upregulated in MSC-3). *MOB4* was one of the most upregulated genes in MSC-3 (Fig. 3*H*). MOB4 participates in STRIPAK complexes that regulate a variety of processes including cell growth, differentiation, signaling, proliferation, and apoptosis. Pathway analysis with the total list of DEG indicates fewer differences between the MSC-1 and MSC-3 subpopulations (Fig. 3*I*). “G2m checkpoint” genes were upregulated in MSC-3 compared with MSC-1 (Fig. 3*I*) and compared with MSC-2 (Supplemental Fig. S3*B*) suggesting that MSC-3 cells are in cell cycle arrest.

### Identification of Depot-Specific ADSC Populations In Vivo

**Figure 4.**
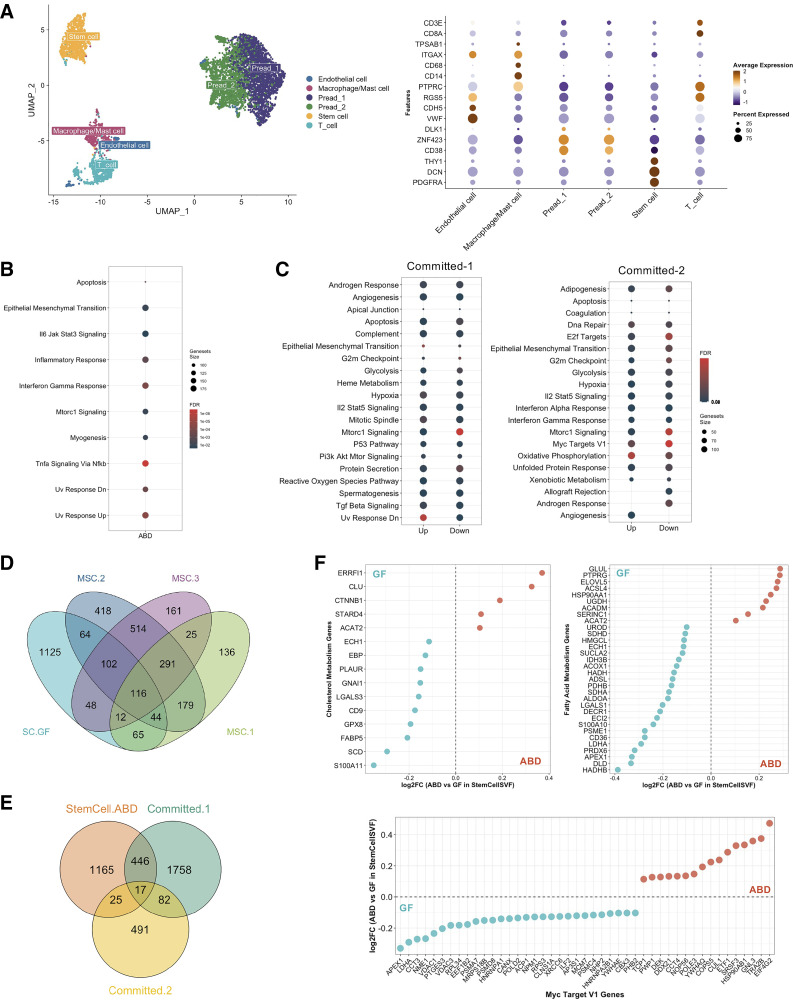
Comparison with in vivo data. UMAP of 5,817 stroma vascular fraction (SVF)-cells from abdominal (ABD) and gluteofemoral (GF) subcutaneous white adipose tissue of five healthy women and dot-plot showing expression of selected marker genes used to label the six clusters (*A*). Dot-plot showing the significant pathways that are upregulated in ABD vs. GF depot for the stem cell cluster, FDR < 0.05, no pathways were identified in GF depot (*B*). Dot-plot showing the significant pathways that are up- and downregulated in Committed-1 and Committed-2 clusters vs. all the other adipose-derived stem cell (ADSC)-clusters, FDR < 0.05 (*C*). Venn diagram displaying the number of overlapping genes that were upregulated in the stem cell (SC) cluster in GF and in each mesenchymal stem cell (MSC)-ADSC clusters (*D*). Venn diagram displaying the number of overlapping genes that were upregulated in the stem cell cluster in ABD and in each committed-ADSC clusters (*E*). Comparison of differentially expressed genes in MSC-2 and MSC-3 in relation to differences between ABD and GF stem cell cluster (*F*). Blue dots mean genes are upregulated in GF stem cells and red dots mean genes are upregulated in ABD stem cells.

We next wanted to know if the subsets of ADSCs we identified in vitro could be found in vivo. We isolated the stroma vascular fraction (SVF) from paired subcutaneous ABD- and GF adipose tissue of five healthy women and performed scRNA-seq directly. Using known marker genes, we identified two preadipocyte populations, characterized by the upregulation of *ZNF423*, *CD38*, and *DLK1*, and one stem cell population, identified by upregulation of *PDGFRA*, *DCN*, and *THY1* (Fig. 4*A*). The number and proportion of these three populations were similar between both depots (Supplemental Fig. 4*A*). We performed DEG analysis on the stem cell population between ABD and GF depot and ran a pathway analysis. We identified 1,029 DEG, the majority of them being upregulated in the ABD depot (879 upregulated in ABD vs. 150 upregulated in GF; Supplemental Fig. S4*B*). Pathway analysis revealed that the genes increased in ABD stem cells were involved in apoptosis, EMT, inflammatory response, INFγ response, Mtorc1 signaling, and Uv response Dn (Fig. 4*B*). We found similar pathways upregulated in ADSC Committed-1 (Fig. 4*C*) and Committed-2 (Fig. 4*D*) clusters, both being ABD-specific ADSC clusters.

We intersected the lists of DEGs upregulated in GF stem cells with the list of DEGs defining MSC-1, MSC-2, and MSC-3. Twenty-eight percent of the total genes upregulated in GF stem cells from the SVF were also upregulated in at least one of the MSC clusters found in cultured ADSCs (Fig. 4*E*). We next intersected the lists of DEGs upregulated in ABD stem cells with the list of DEG defining Committed-1 and Committed-2 clusters. Twenty-nine percent of the total genes upregulated in ABD stem cells from the SVF were also upregulated in at least one of the committed clusters found in culture ADSCs (Fig. 4*F*). Together, this provides strong evidence that the cultured cell clusters map onto clusters present in fresh adipose tissue.

Next, we selected the 50 most upregulated genes in four clusters that are relevant when studying ADSC function, i.e., were differentially regulated between Committed and MSC clusters (respectively ABD and GF specific; Fig. 2*D*): “Myc Target V1,” “Adipogenesis,” “Cholesterol Metabolism,” and “Fatty Acid Metabolism.” Then we intersected these lists with the differentially expressed genes between ABD- and GF SVF stem cell populations. This analysis highlighted the genes that were depot-specific in both datasets. Because all the pathways we chose were found upregulated in MSC subpopulations (Fig. 2*D*), we expected to see a higher overlap with genes increased in GF stem cells compared with ABD stem cells. The dot-plots in Fig. 4*F* and Supplemental Fig. S4*C* show the fold change of expression between ABD and GF depot in stem cells (from SVF) for these genes. For the four pathways studied, we identified an important overlap between the two data sets with a higher number of genes upregulated in GF stem cells (negative FC, blue dots), specifically for genes involved in cholesterol, FA metabolism, and Myc Target V1 (Fig. 4*F*). Altogether these results suggest that the depot-specific differences observed in cultured ADSCs are also observed in the matching freshly isolated SVF cells that validates our studies on the cultured SVF.

### ADSC Subpopulations in Metabolic Conditions

**Figure 5.**
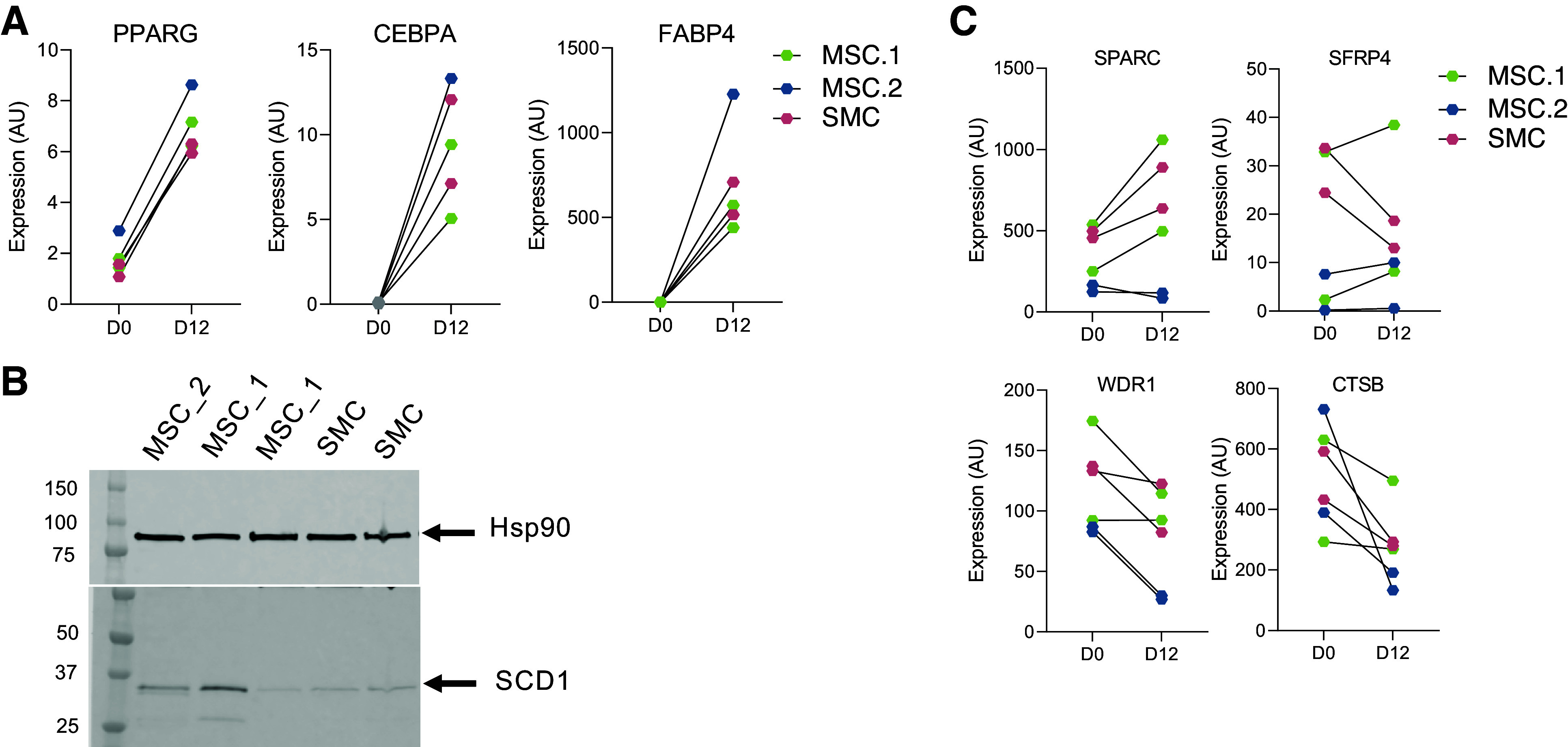
Validation in vitro of the single-cell (sc)RNA-seq findings. Gene expression levels of adipogenic marker genes before (*D0*) and after differentiation (*D12*) in mesenchymal stem cell (MSC)-1, MSC-2 and SMC previously fluorescence-activated cell sorting (FACS) sorted (*A*). The colors describe the different subpopulations of adipose-derived stem cell (ADSC) isolated by FACS. Lines are drawn connecting values from before and after differentiation. Protein expression levels of adipocyte marker (SCD1) was measured on *day 12* of differentiation of MSC-1, MSC-2 and SMC. Hsp90 were used as internal control (*B*). Gene expression levels of fibrotic and insulin resistant marker genes before (*D0*) and after differentiation (*D12*) (*C*). The colors describe the different subpopulations of ADSC isolated by FACS. Lines are drawn connecting values from before and after differentiation.

Next, we investigated whether the proportion of ADSC subpopulations specific to ABD or GF was associated with body shape. The UMAP repartition of the ABD ADSCs (Fig. 5*A*) and GF ADSCs (Fig. 5*B*) in apple and pear subjects showed differences in the cell numbers per clusters. Remarkably, Committed-1 cluster was slightly higher in apple-ABD ADSCs compared with ABD-pear ADSCs (Fig. 5*A*) consistent with our hypothesis that there is a defect in differentiation in ABD-AT isolated from apple-shaped women. In GF depot, we noticed the absence of the Committed-1 and Committed-2 subpopulations in apple women (Fig. 5*B*). The absence of stem cells committed to the adipocyte lineage in this depot for these women is also consistent with the hypothesis that adipose differentiation and lipid accumulation in cells from the apple GF depot have a reduced capacity to expand and store excess lipid. Finally, MSC-1 population was increased in pear compared with apple subjects in ABD depot (Fig. 5*A*) and was also increased in the apple samples compared with pear subjects in GF depot (Fig. 5*B*; *P* = 0.04). The ABD depot in pear-shaped women and the GF depot in apple-shaped women are both smaller compared with their paired SAT (GF and ABD, respectively). These findings further align with our overall hypothesis that predicts there is a reduced capacity for MSC-1 to differentiate into adipocytes.

**Figure 6.**
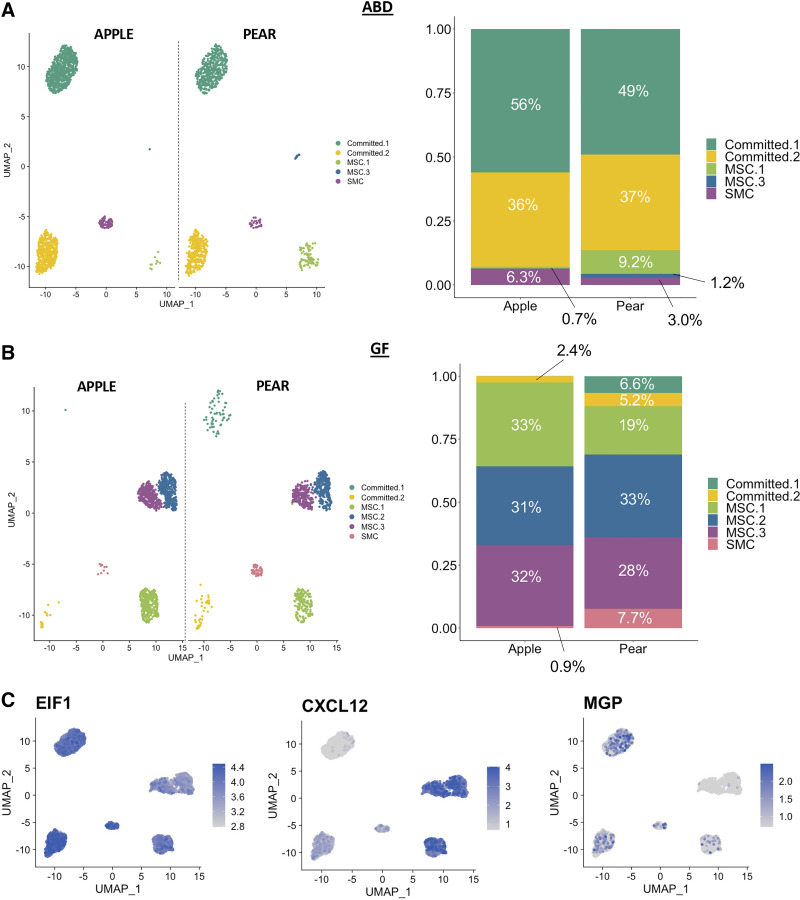
Adipose-derived stem cell (ADSC) clusters in metabolic conditions. UMAPs and stacked bar-plots showing the repartition of the ADSCs subpopulations according to the body shape (apple vs. pear) in abdominal (ABD) depot (*A*) and gluteofemoral (GF) depot (*B*). Feature plot showing expression of *EIF1*, *CXCL12,* and *MGP* genes (*C*).

We then compared our data with a study that identified genes dysregulated in type 2 diabetes (T2D) adipose progenitor cells by Vijay et al. (11). They analyzed human SVF by scRNA-seq on the 10× genomics platform and found 10 genes significantly differentially regulated between non-T2D and T2D. Among those, we identified three genes with differential expression in our ADSC subpopulations (Fig. 6*C*). *EIF1* was upregulated in Committed-2 versus Committed-1 and in MSC-1 versus MSC-2/MSC-3. *CXCL12* was upregulated in Committed-2, whereas *MGP* was upregulated in Committed-1 and in MSC-1.

The recent work from Emont et al. (10) described six subpopulations of human adipose progenitor cells (APCs) in subcutaneous (ABD) and visceral adipose tissue using 10× genomic scRNA-seq technology. We looked at the expression of the gene markers highlighted in their study in our subpopulations of ADSCs. Remarkably, PDE4D, marker gene of an APC subpopulation found more abundant in VAT, was upregulated in the Committed-1 ADSC subpopulation in our study (Supplemental Fig. S5). Committed-1 ADSCs are potentially less capable of differentiation and are more abundant in apple individuals. We also observed higher expression of EPHA3 in Committed-1 ADSC (Supplemental Fig. S5), this is a gene that defines another subpopulation of human APC in VAT and SAT that likely represents the antiadipogenic Areg population reported by Schwalie et al. (50). These observations are consistent with the lower capacity of VAT precursor cells to differentiate and suggest that the ABD cells in apple-shaped women share similarity with the VAT. In contrast, ALDH1A3, marker gene of early multipotent progenitor cells reported by Emont et al., was more expressed in the three subpopulations enriched in GF adipose depot and that we named MSC (Supplemental Fig. S5). This result confirms that GF precursors are less engaged in the adipocyte lineage compared with the ABD cells.

### “SMC” and “MSC-1” Subpopulations Have Reduced Adipogenic Potential In Vitro

Our scRNA-seq data uncovered six distinct cell populations with specific transcriptional profiles potentially associated with variation in adipogenic differentiation capacity. To validate this hypothesis, we compared the in vitro differentiation capacity of a subset of these cell clusters. To do this, we selected candidate markers that were the most enriched in the MSC-1, MSC-2, and SMC subpopulations and used these markers to purify the specific clusters using fluorescence-activated cell sorting (FACS) from the initial pool of ABD- and GF ADSCs. The markers used for each selection are described in the materials and methods and their expression in each cluster of ADSC is shown in Supplemental Fig. S6.

Because the SMC are often considered contaminants in cultured ADSCs, and the MSC-1 are enriched in GF-apple samples, we tested the capacity of the MSC-1, MSC-2, and SMC subpopulations to differentiate in the presence of a submaximal adipogenic differentiation cocktail (see materials and methods). Interestingly, the SMC and MSC-1 subpopulations differentiated to a lesser degree, reflected by their lower expression of *PPARG*, *CEBPA*, and *FABP4* at the end of the differentiation period (*day 12*) in these cells compared with the MSC-2 cells (Fig. 6*A*). Using Western blotting, we measured the abundance of SCD1 [enzyme promoting long-chain fatty acid desaturation in white adipose tissue (WAT)] after 12 days of differentiation of the MSC-1, MSC-2, and SMC. SCD1 expression was lower in the SMC, whereas one of the fully differentiated MSC-1 samples had comparable levels with the MSC-2 cells (Fig. 6*B*). Interestingly, after differentiation, some fibrotic and insulin resistance markers (*SPARC1*, *SFRP4*, *WDR1*, and *CTSB*) were still more expressed in SMC and MSC-1 compared with MSC-2 (Fig. 6*C*). These results suggest that the cells retained some of their MSC-like transcriptome identity in vitro during the process of maturation in culture.

These two findings are consistent with our global hypothesis that in apple-shaped women, there is a failure to fully expand their GF depot to store excess lipids below the waist. The MSC-1 showed also a fibrotic transcriptional profile. This profile was maintained during in vitro differentiation (Fig. 6*C*) and may contribute to MSC-1’s lower ability to mature into healthy lipid-storing adipocytes and their increased propensity for fibrosis in vivo.

## DISCUSSION

In conclusion, to our knowledge, our study reveals for the first time, distinct subpopulations of ADSCs in ABD- and GF adipose tissue depots, each defined by a distinctive transcriptome profile. These findings support the concept that the stem cell repertoire is related to the well-known functional and structural differences in subcutaneous adipose function. It also emphasizes that in vitro results can be significantly influenced by the original anatomic location of the subcutaneous adipose tissue collected for analysis. Additional experiments will be necessary to fully understand and map these different subpopulations of ADSCs onto the well-known functional differences of mature adipocytes. ABD-specific ADSCs were characterized by an increased expression of committed adipocyte lineage marker genes, whereas the GF-ADSCs were enriched for genes involved in cell growth, FA metabolism, and cholesterol metabolism. Additional comparisons between the individual clusters suggested that one of the ABD-ADSC clusters and one of the GF-ADSC clusters were less capable of differentiation.

The GF precursor cells have higher expression of cholesterol and fatty acid storage genes, suggesting that these cells could handle more lipid storage than the ABD depot and could participate in the healthy accumulation of fat in the lower body. On the opposite, the higher expression of genes involved in extracellular matrix (ECM) in ABD-ADSCs suggests that these cells would differentiate less and in consequence limit the expansion of the ABD depot in favor of adipocyte hypertrophy, known to be associated with ectopic fat deposition and insulin resistance. It is also possible that the unique microenvironments of each subcutaneous adipose tissue depot (ABD vs. GF) determine the specific subpopulations of ADSCs populating these fat depots. Our study does not allow us to determine if the subpopulations of ADSCs we described are a cause or a consequence of the regional differences in obesity. Xenograft experiments, among others, using isolated human SVF from ABD and GF, could address this important question.

Our studies also reveal for the first time, that the relative proportion of ADSC subtypes differs in apple-shaped versus pear-shaped women; this was particularly evident in the GF depot. In apple-shaped women, GF-ADSCs contained a higher proportion of a unique MSC population, defined by a transcriptome with increased expression of genes involved in proliferation (MSC-1). Further analysis using FACS selection to isolate this subpopulation demonstrated that these cells potentially differentiate less in vitro than the other MSC subpopulations. The apple-ABD depot was also depleted of the “committed” stem cell populations, suggesting a defect of differentiation in this particular depot for apple-shaped women. The idea that adipose stem cell precursors might ultimately determine how adipose tissue expands differently in the various regions of the body is an important orthogonal part of the “expandability hypothesis” of metabolic disease (51), which is supported by our analyses. Here, we showed evidence that the stem cell repertoire may be a key part of the adipose tissue expandability theory in apples versus pears and our data support our global hypothesis that a deficit in lower body adipose tissue expansion in apple-shaped women, leads to an unhealthy and inappropriate storage of fat in their upper body, potentially through the accumulation of proliferative stem cells that are less capable of lipid accumulation.

A major limitation of our study is the use of cultured cells. First, the cells were cryopreserved and thawed, processes that could induce cell loss. To minimize these effects, we used a minimum quantity of DMSO to cryopreserve the cells (10% DMSO) and a fast-thawing method that has been proved to reduce cell loss and showed optimal results with adipose tissue precursor cells culture (52, 53). In vitro conditions most certainly modified the transcriptome of the ADSCs as compared with the native in vivo environment. However, our experiments comparing cultured cell sequencing to fresh SVFs showed that many of the differential properties identified in the cultured ADSCs are present in freshly isolated SVF cells. This provides strong initial validation of the utility and validity of cultured cells using our specific methodologies. Even in this in vitro data set, we still identify differences in the proportion of subpopulations of ADSCs across body shape indicating that distinct subpopulations exist early in the development of ABD and GF adipose tissue depots. Because these two depots are genetically identical, we speculate that specific epigenetic mechanisms underlie their distinct transcriptomes. In fact, our recent studies provide strong supportive evidence for both differential open-chromatin and positive and negative histone modifications in chromatin isolated from ABD versus GF and apple versus pear subjects (16, 19).

Taken together, these data provide a solid foundation for research to further explore the epigenome of cultured ADSCs to better understand their heterogeneity, and the impact on adipose depot expansion that may ultimately help explain the underlying molecular mechanisms that lead to the known differential risk of apples versus pears for cardio-metabolic disease. These data are also fundamental to study the importance of adipose tissue function and dysfunction in diseases and conditions such as polycystic ovary syndrome (PCOS), T2D, and during aging.

## DATA AVAILABILITY

scRNA-seq data sets have been deposited at GEO, under GEO No. GSE209618 and GEO No. GSE255996 and are publicly available as of the date of publication.

## SUPPLEMENTAL DATA

10.6084/m9.figshare.24911475 Supplemental Figs. S1–S6 and Supplemental Table S1: https://doi.org/10.6084/m9.figshare.24911475.

## GRANTS

This study was supported by NIH Grant/Award No. R01DK107009 (to Steven R. Smith).

## DISCLOSURES

No conflicts of interest, financial or otherwise, are declared by the authors.

## AUTHOR CONTRIBUTIONS

A.D. conceived and designed research; A.D. and M.E.H. performed experiments; A.D., K.L.W., and L.H. analyzed data; A.D., K.L.W., and S.R.S. interpreted results of experiments; A.D. and K.L.W. prepared figures; A.D. drafted manuscript; A.D., K.L.W., L.H., L.M.S., T.F.O., and S.R.S. edited and revised manuscript; A.D., K.L.W., L.H., L.M.S., T.F.O., and S.R.S. approved final version of manuscript.
